# Supplementary figures and images for: The transcription factor HBP1 promotes ferroptosis in tumor cells by regulating the UHRF1-CDO1 axis
Source: PLoS Biol. 2023 Jul 5;21(7):e3001862. doi: 10.1371/journal.pbio.3001862 (PMC10351698; doi:10.1371/journal.pbio.3001862)

**A**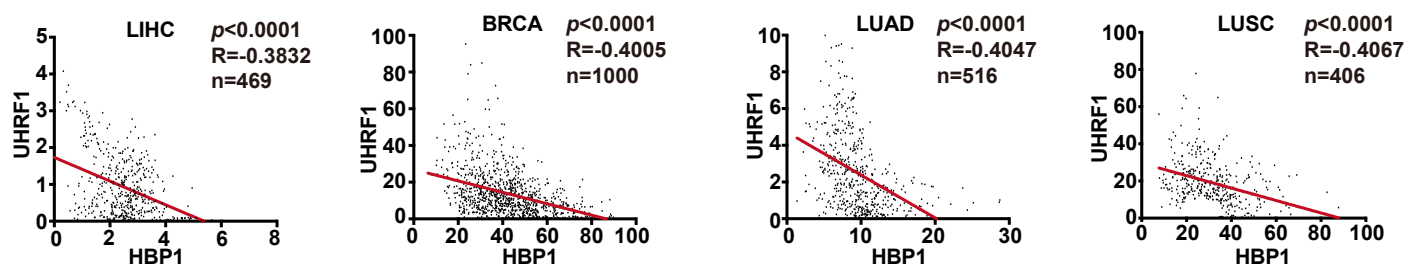**B**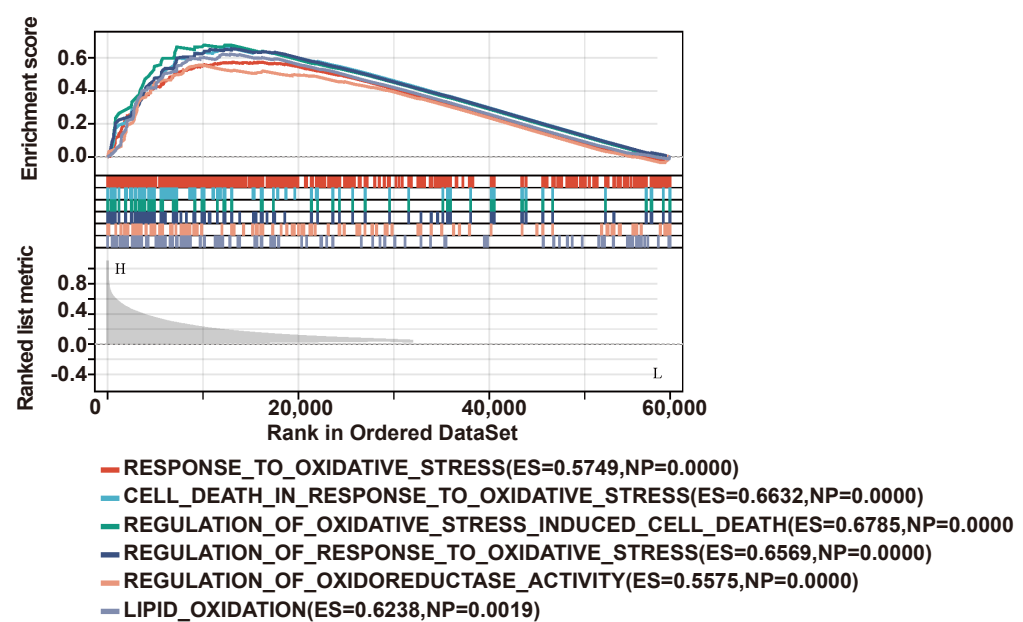

Supplement: S1 Fig — (A) HBP1 negatively correlated with UHRF1 mRNA levels in the TCGA cohort of LIHC, BRCA, LUAD, and LUSC. The linear relationship was determined by a Pearson correlation analysis. (B) GSEA plots of genes in high HBP1 expression group compared with low HBP1 expression group. High-rank gene sets are shown with the ES, normalized ES, and nominal p valve. The underlying data for S1A and S1B Fig can be found in S1 Data. BRCA, Breast cancer; ES, enrichment score; GSEA, Gene Set Enrichment Analysis; HBP1, HMG box-containing protein 1; LIHC, liver hepatocellular carcinoma; LUAD, lung adenocarcinoma; LUSC, lung squamous cell carcinoma; TCGA, The Cancer Genome Atlas; UHRF1, ubiquitin-like with PHD and RING finger domains 1. (PDF) [file pbio.3001862.s001.pdf]

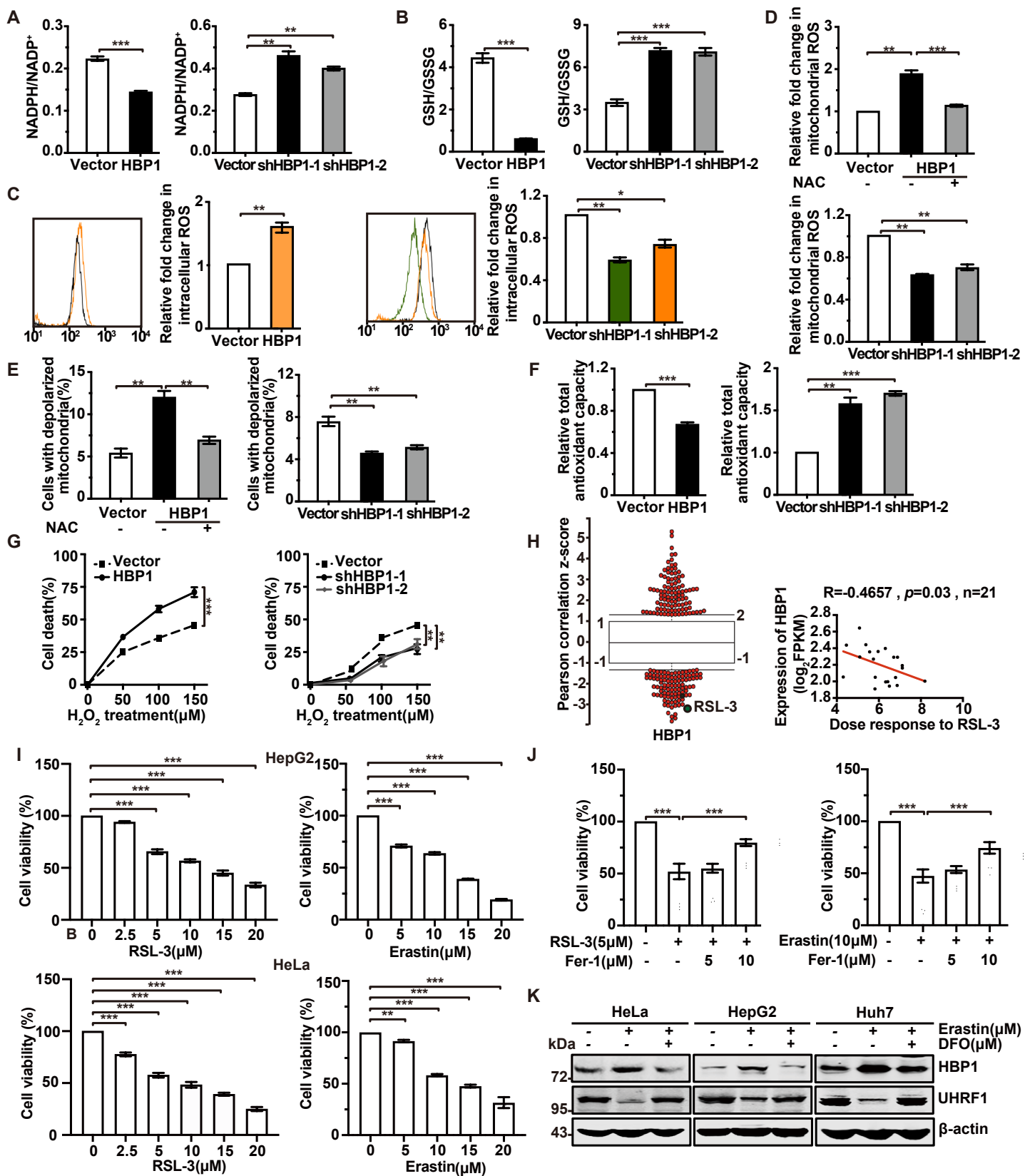

Supplement: S2 Fig — (A) NADPH/NADP+ ratio and (B) GSH/GSSG ratio were measured in the indicated cells. (C) Intracellular ROS of the indicated cells were stained by DCFH-DA and determined by FCM. (D) Mitochondrial ROS were stained by MitoSOX Red and measured by fluorescence microplate reader. The fold changes of ROS levels relative to controls were shown. (E) The cells with depolarized mitochondria are represented as the cells that have lost ΔΨm. The proportions of the cell with depolarized mitochondria in the indicated cells are shown. For NAC treatment in (D) and (E), HepG2/HBP1 cells were pretreated with 100 nM NAC for 24 h before collection. (F) Total antioxidant capacity in the indicated cells was detected. (G) Cell death was determined in the indicated cell with treatment of the incremental doses of H2O2 for 24 h. (H) Calculation of z-scored Pearson correlation coefficients between small-molecule sensitivity data, expressed as AUCs, with basal gene-expression measurements, expressed as log2 robust-multi-array-average values. Green dot means the expression level of HBP1 gene was correlated with the sensitivity of RSL-3 (left panel). Correlation between HBP1 expression and RSL-3 sensitivity, based on the liver cancer cell lines (n = 21) from CTRP. Dose responses are normalized AUC values. The linear relationship was determined by a Pearson correlation analysis (right panel). (I) Viability of HeLa and HepG2 cells treated with different concentrations of Erastin or RSL-3. (J) HeLa cells were treated with Erastin or RSL3 in the absence or presence of ferrostatin-1 (5 μM or 10 μM) for 24 h, and then cell viability was measured. (K) Protein levels of HBP1 and UHRF1 in HeLa, HepG2, and Huh7 cells were treated with Erastin (10 μM) alone or in combination with DFO (50 μM). The underlying data for S2A–S2J Fig can be found in S1 Data. Error bars represent S.D. *, p < 0.05, **, p < 0.01, ***, p < 0.001. AUCs, areas under concentration–response curves; CTRP, Cancer Therapeutics Response Portal; [file pbio.3001862.s002.pdf]

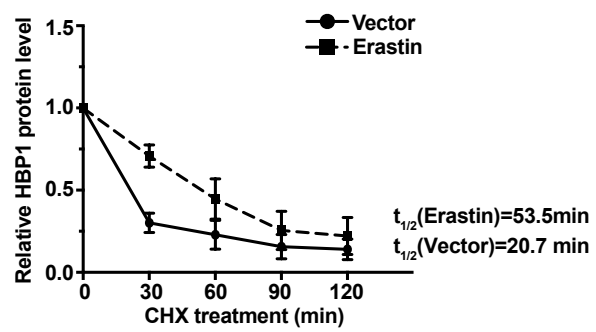

Supplement: S3 Fig — HeLa cells were treated with Erastin for 24 h, and cells were incubated with the protein synthesis inhibitor CHX for 0, 30, 60, 90, or 120 min before collect. HBP1 and protein levels were detected by western blotting. Quantification of HBP1 protein levels was determined using Image J software normalized to β-actin. The underlying data for S3 Fig can be found in S1 Data. CHX, cycloheximide; HBP1, HMG box-containing protein 1. (PDF) [file pbio.3001862.s003.pdf]

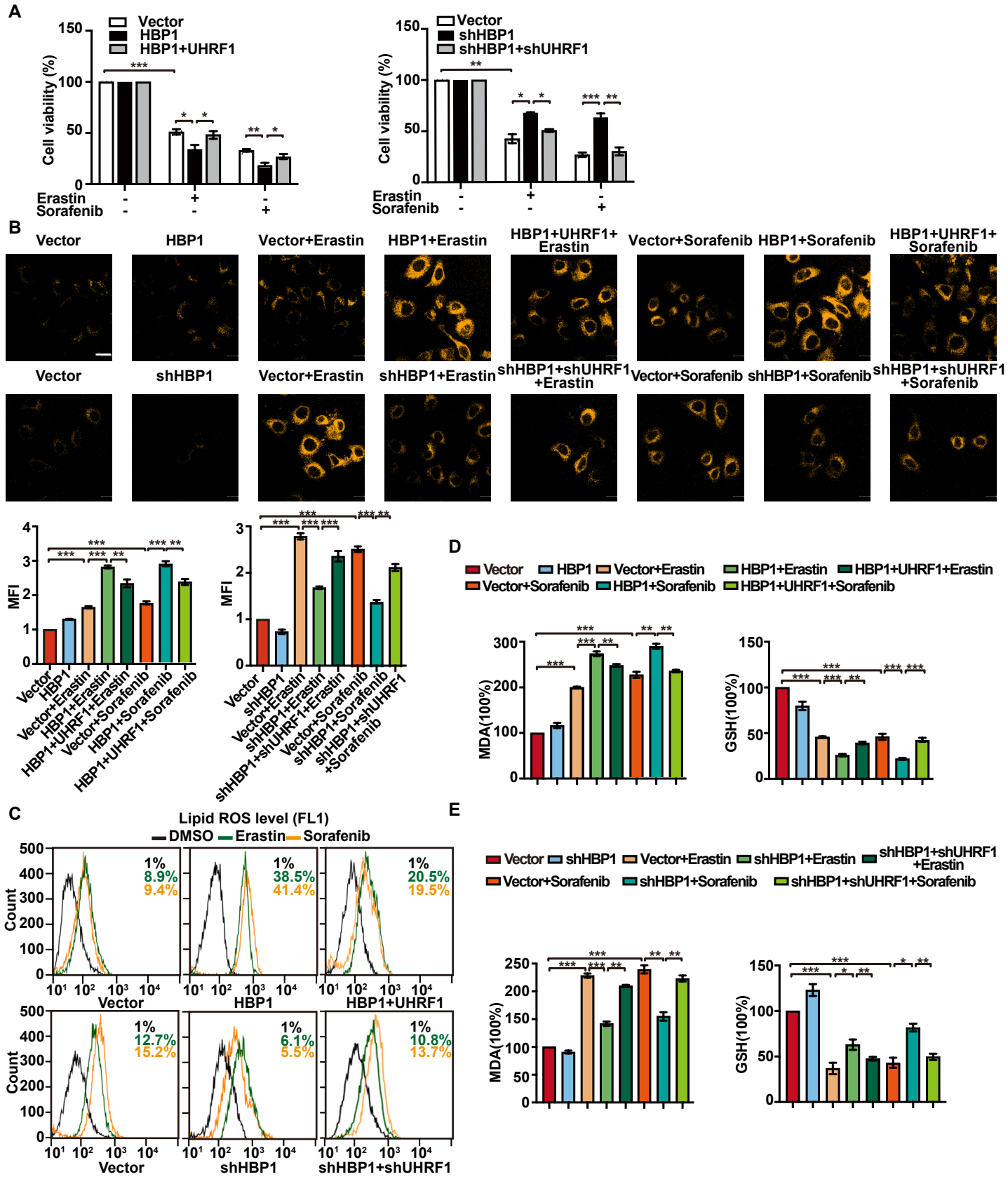

Supplement: S4 Fig — (A) Cell viability was conducted with HepG2 cells stably transfected with vector, HBP1, HBP1+UHRF1 or vector, shHBP1, shHBP1+shUHRF1. (B, C) Indicated cells were treated with or without 10 μM Erastin/10 μM Sorafenib for 24 h. Cells were collected, and confocal was used to detect Fe2+ levels and flow cytometry was used to detect BODIPY-C11 fluorescence signal for ROS. (D, E) Indicated cells were lysed and MDA content and GSH content were measured. Scale bar = 10 μm. The underlying data for S4A, S4B, S4D and S4E Fig can be found in S1 Data. Differences between 2 groups were calculated using a two-tailed Student t test. One-way ANOVA was performed to assess differences among multiple groups. Error bars represent S.D. *, p < 0.05, **, p < 0.01, ***, p < 0.001. GSH, glutathione; HBP1, HMG box-containing protein 1; MDA, malondialdehyde; ROS, reactive oxygen species; UHRF1, ubiquitin-like with PHD and RING finger domains 1. (PDF) [file pbio.3001862.s004.pdf]

A

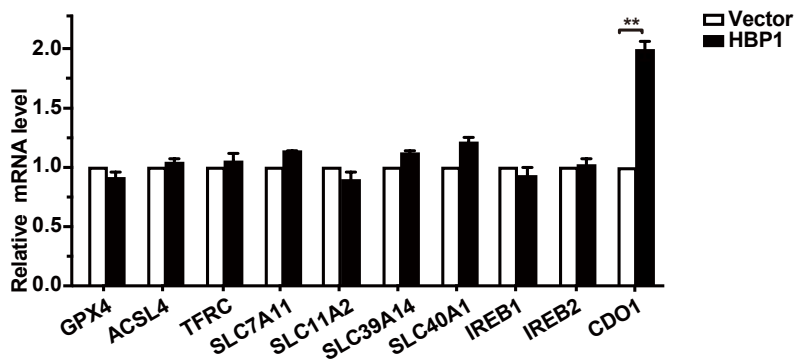

B

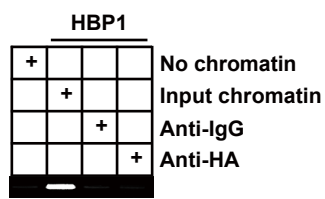

Supplement: S5 Fig — (A) Quantitative RT-PCR showing mRNA expression of ferroptosis-related genes in HBP1 overexpressed cells. (B) HBP1 did not binding to the endogenous CDO1 promoter. ChIP assays were used to test the binding of exogenous HBP1 to endogenous CDO1 gene. HEK293T cells were transfected with HA-HBP1. The region from position −456 to position −658 contains the predicted HBP1 affinity site and was analyzed by specific PCR. Anti-HA antibody was used in the indicated lanes. The underlying data for S5A Fig can be found in S1 Data. CDO1, cysteine dioxygenase 1; ChIP, chromatin immunoprecipitation; HBP1, HMG box-containing protein 1; RT-PCR, real-time PCR. (PDF) [file pbio.3001862.s005.pdf]

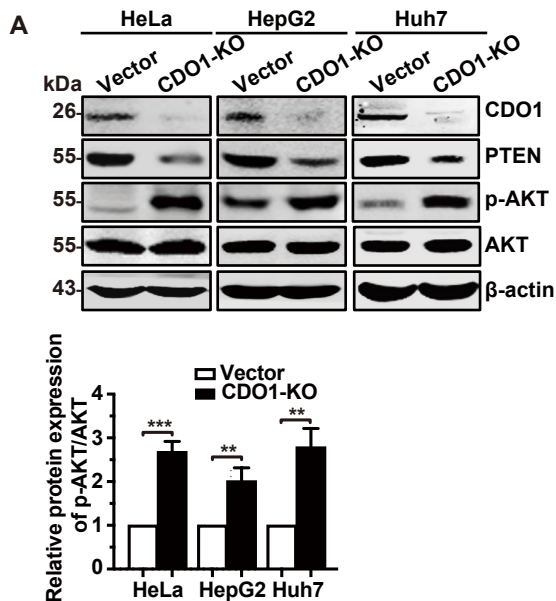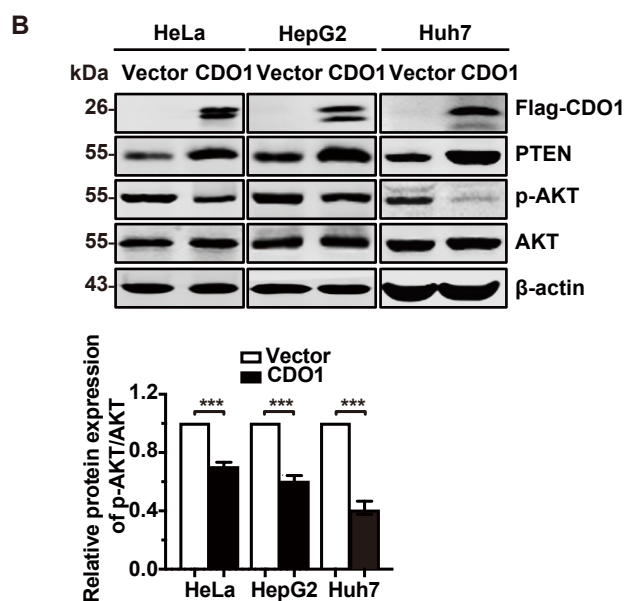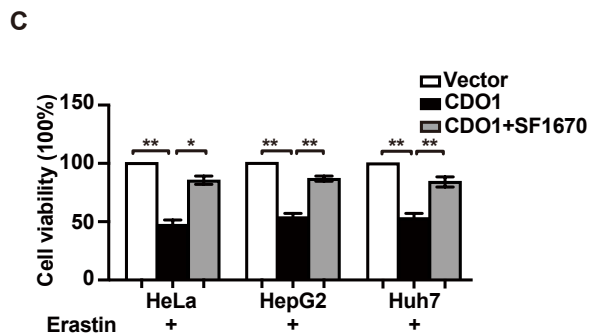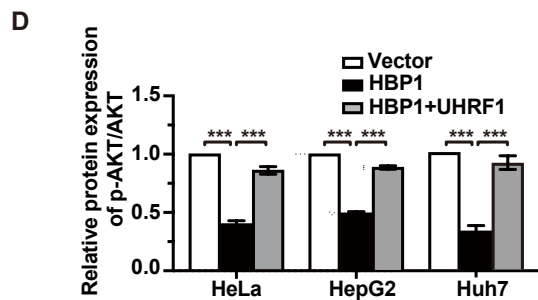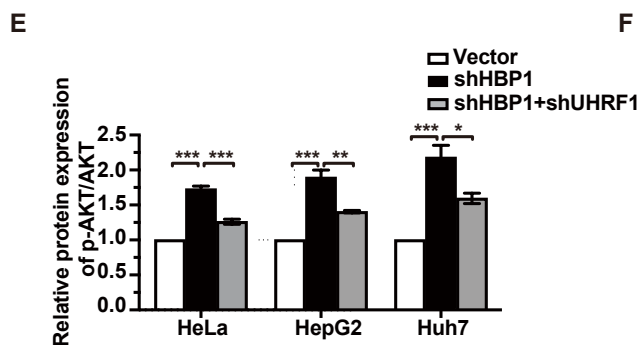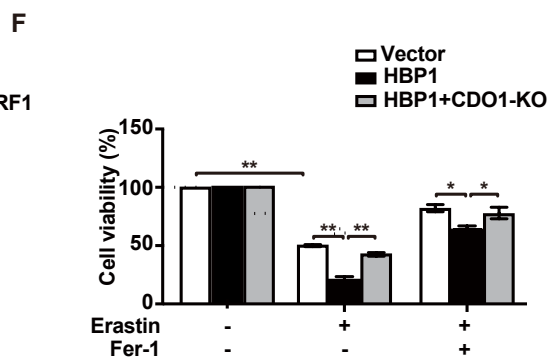

Supplement: S6 Fig — (A) CDO1 knockout promotes cell proliferation. HeLa, HepG2, and Huh7 cells with CDO1 knockout were analyzed by western blotting to detect the protein levels of CDO1, PTEN, p-AKT, and AKT. (B) CDO1 overexpression inhibits cell proliferation. HeLa, HepG2, and Huh7 cells with CDO1 overexpression were analyzed by western blotting to detect the protein levels of CDO1, PTEN, p-AKT, and AKT. (C) CDO1 can partially promote ferroptosis through PTEN-AKT signal pathway. HeLa, HepG2, and Huh7 cells with CDO1 overexpression treated with Erastin (10 μM) and SF1670 (10 μM) for 24 h. Cell viability was measured by MTT. (D, E) Indicated cells p-AKT/AKT protein ratio was determined using Image J software. (F) Indicated cells were treated with or without 10 μM Fer-1 for 24 h in the presence of Erastin (10 μM). Cell viability was measured using MTT. The underlying data for S6A–S6F Fig can be found in S1 Data. CDO1, cysteine dioxygenase 1; Fer-1, Ferrostatin-1; HBP1, HMG box-containing protein 1; p-AKT, phospho-AKT; UHRF1, ubiquitin-like with PHD and RING finger domains 1. (PDF) [file pbio.3001862.s006.pdf]

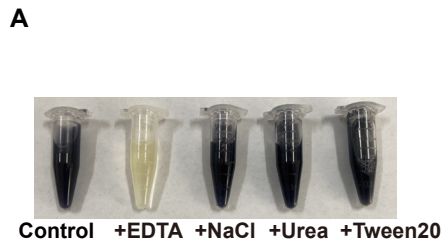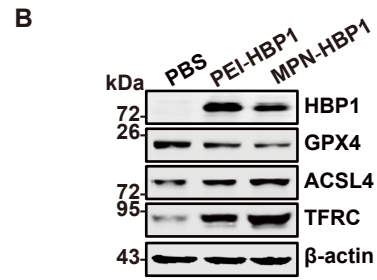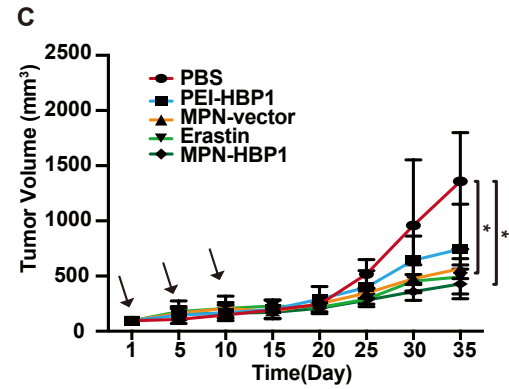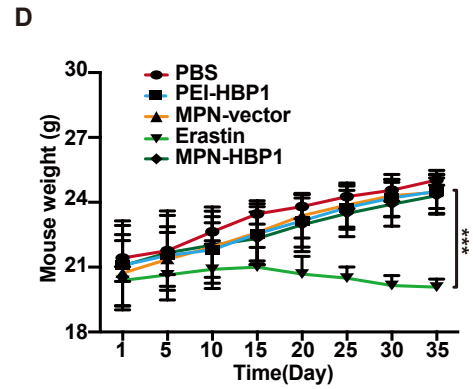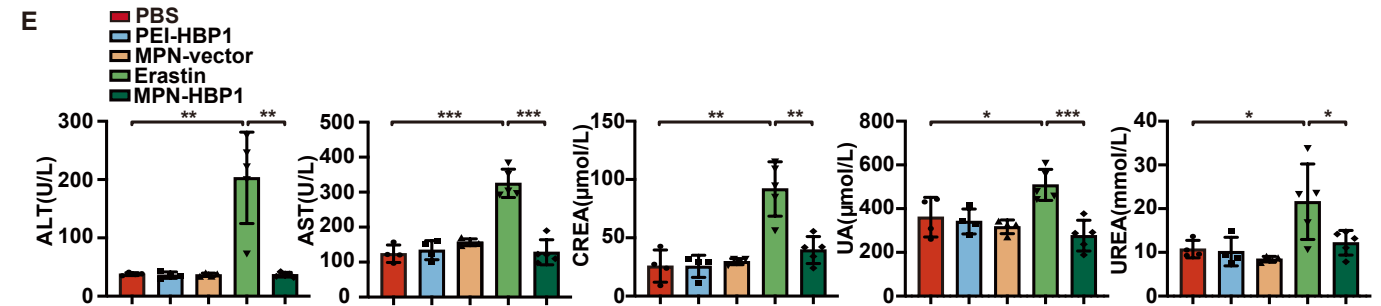

Supplement: S7 Fig — (A) MPN-HBP1 dissociation by EDTA, NaCl, urea, and Tween 20. MPN-HBP1 was effectively dissociated by EDTA due to complexation competition. (B) HBP1, GPX4, ACSL4, and TFRC protein levels of PBS, PEI-HBP1, and MPN-HBP1 treated HeLa cells. (C, D) Tumor volume curves and tumor weight curves of experimental mice at the first 35 days. (E) Blood biochemistry indexes of experimental mice after IV injected with PBS, PEI-HBP1, Erastin, MPN-vector, and MPN-HBP1. The underlying data for S7C–S7E Fig can be found in S1 Data. ACSL4, acyl-CoA synthetase long chain family member 4; EDTA, ethylene diamine tetraacetic acid; GPX4, glutathione peroxidase 4; HBP1, HMG box-containing protein 1; IV, intravenous injection; MPN, metal polyphenol network; PEI, polyethyleneimine; TFRC, transferrin receptor. (PDF) [file pbio.3001862.s007.pdf]
