# Supplementary material for: The transcription factor HBP1 promotes ferroptosis in tumor cells by regulating the UHRF1-CDO1 axis
Source: PLoS Biol. 2023 Jul 5;21(7):e3001862. doi: 10.1371/journal.pbio.3001862 (PMC10351698; doi:10.1371/journal.pbio.3001862)
Supplement: S1 Table — (DOCX) [file pbio.3001862.s008.docx]

S1 Table. Primers used in the experiment

| **No.1 RT-**  **PCR** | **Gene** | **Forward Primer 5'-3'** | **Reverse Primer 5'-3'** |
| --- | --- | --- | --- |
| **1** | **GAPDH** | **CCATGGAGAAGGCTGGGG** | **CAAAGTTGTCATGGATGACC** |
| **2** | **HBP1** | **TGAAGGCTGTGATAATGAGGAAGAT** | **CATAGAAAGGGTGGTCCAGCTTA** |
| **3** | **UHRF1** | **CCTGGCGTCACTCAAGGA** | **TCCTGACAGCAGATACACTGGA** |
| **4** | **CDO1** | **TCTCTGTTGGGGTGAAGGAC** | **GCCAGGCAAATAATGTCTCC** |
| **5** | **SLC7A11** | **TCTCCAAAGGAGGTTACCTGC** | **AGACTCCCCTCAGTAAAGTGAC** |
| **6** | **GPX4** | **TGGGAAATGCCATCAAGTG** | **GGGGCAGGTCCTTCTCTATC** |
| **7** | **SLC11A2** | **CTGCACCATGAGGAAGAAGC** | **TGGATACCTGAGTGGCTGAGT** |
| **8** | **SLC40A1** | **CCCCAGCTCTAGCTGTGAAA** | **CAGGGGTTTTGGCTCAGTAT** |
| **9** | **IREB1** | **CCTCAGCCCCTGTCAAAA** | **GATTACTGATGGCCACGTGTT** |
| **10** | **IREB2** | **CTGCCGAGGATCTTGTGATTC** | **GGGTGTATTCTCAATCTGCGAA** |
| **11** | **SLC39A14** | **AAGGCCCTACTCAACCACCT** | **CGACTGCTCGCTGAAATTGTG** |
| **12** | **ACSL4 F** | **GCTACTTGCCTTTGGCTCATGTGC** | **GTGTGGGCTTCAGTACAGTACAGTCTCC** |
| **No.2 ChIP** | **UHRF1** | **GTGCAGGTGGTCTCCTAGGG** | **CCCAGCGTGCAAAGGGGCT** |
|  | **CDO1** | **GTTCTTGGTTTTCCATCATTTC** | **AGGCCAGAGAGTGCCTGTTTT** |
| **No.3 MSP** | **CDO1** | **Methylation-specific primers** | **TTTTTGGGACGTCGGAGATAAC** |
|  |  |  | **CGAAAAAACCCTACGAACACG** |
|  |  | **Un-methylation-specific primers** | **GATTTTTGGGATGTTGGAGATAAT** |
|  |  |  | **AAAACAAAAAAACCCTACAAACACA** |
| **No.4 shRNA** | **HBP1sh-1** | **5′-ACTGTGAGTGCCACTTCTC-3′** |  |
|  | **HBP1sh-2** | **5’-CACATGGAGCTTGATGACC-3’** |  |
|  | **UHRF1sh-1** | **5'-TGGTCAATGAGTACGTCGATTTCAAGAGAATCGACGTACTCATTGACCTTTTTTC-3′** |  |
|  | **UHRF1sh-2** | **5’-TGCCTTTGATTCGTTCCTTCTTTTCAAGAGAAAGAAGGAACGAATCAAAGGCTTTTTTC-3’** |  |
