# Supplementary material for: The transcription factor HBP1 promotes ferroptosis in tumor cells by regulating the UHRF1-CDO1 axis
Source: PLoS Biol. 2023 Jul 5;21(7):e3001862. doi: 10.1371/journal.pbio.3001862 (PMC10351698; doi:10.1371/journal.pbio.3001862)

Fig.1C

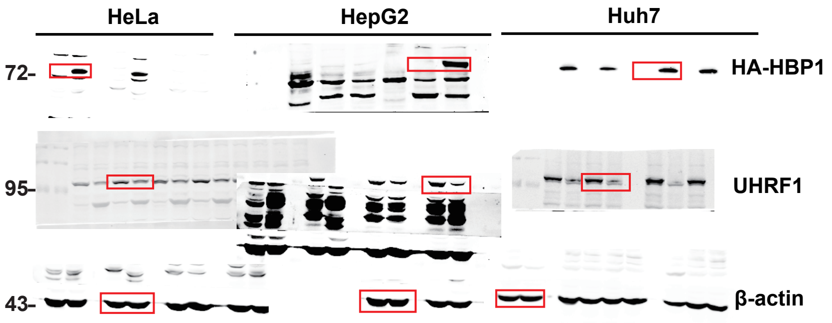
HeLa
HepG2
Huh7
72-
HA-HBP1
95-
UHRF1
43-
β-actin

Fig.1D

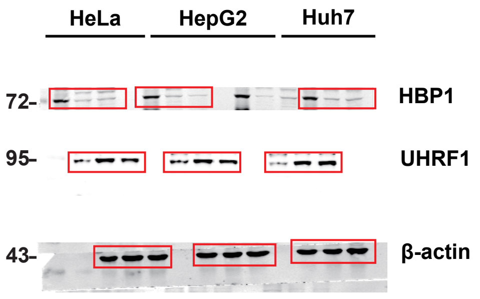
HeLa
HepG2
Huh7
72-
HBP1
95-
UHRF1
43-
β-actin

Fig.2D

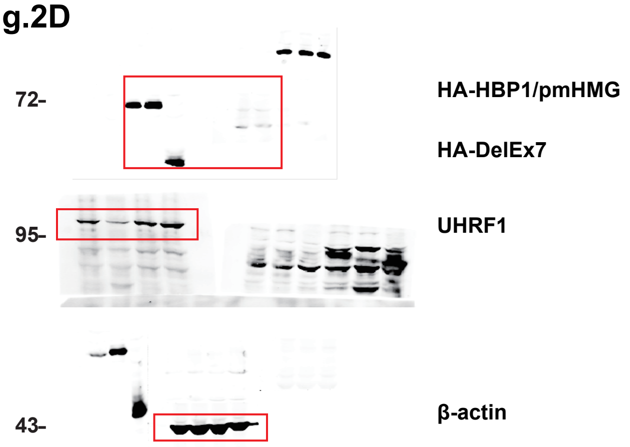

Fig.2E

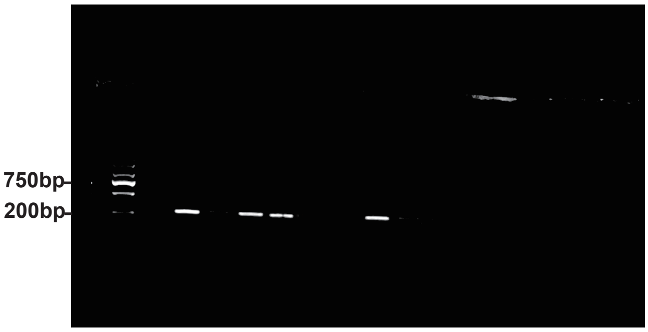

Fig.3A

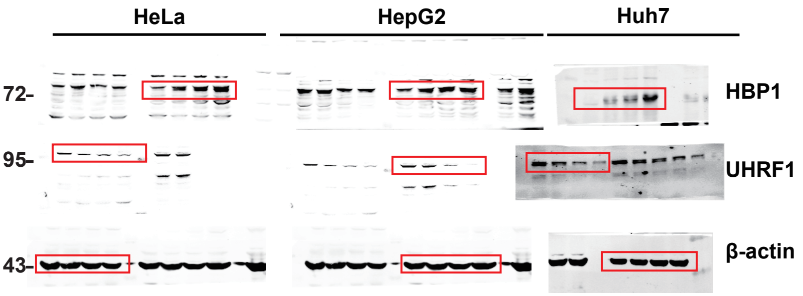

Fig.3B

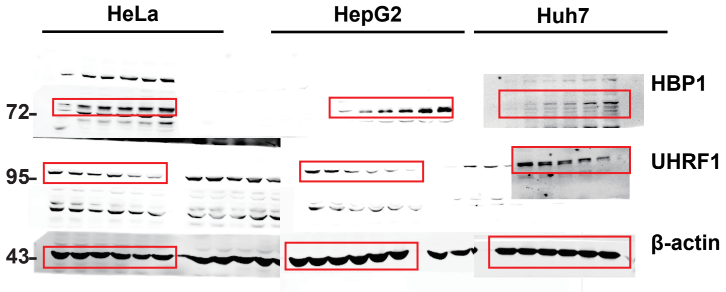

Fig.3C

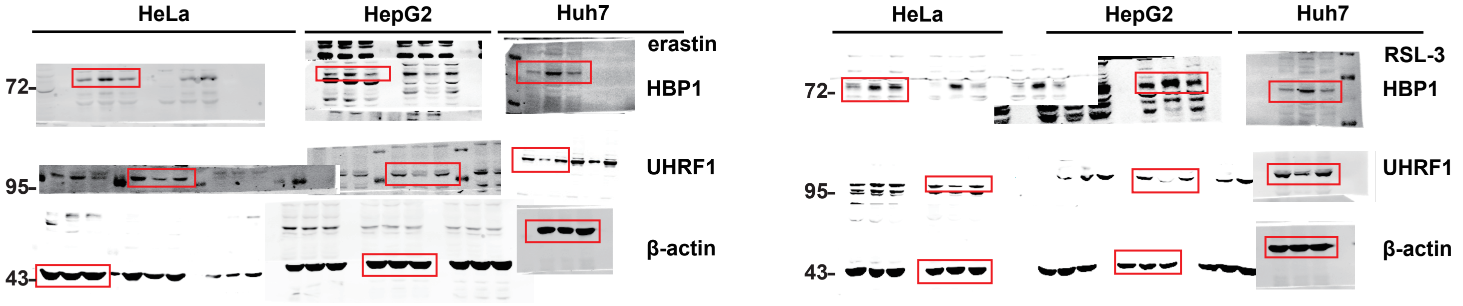

Fig.3D

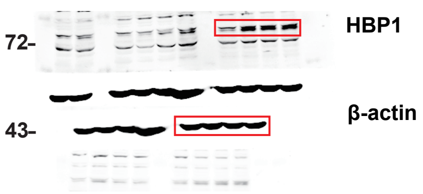

Fig.3E

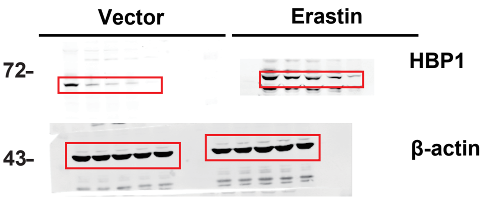

Fig.3F

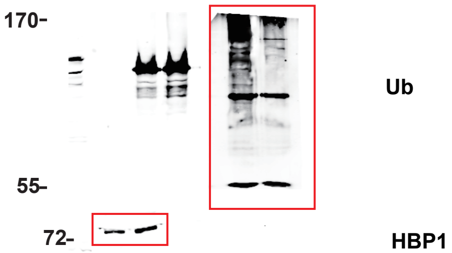

Fig.3G

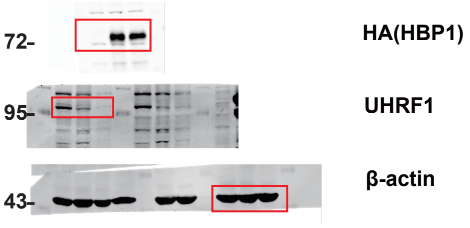

Fig.3J

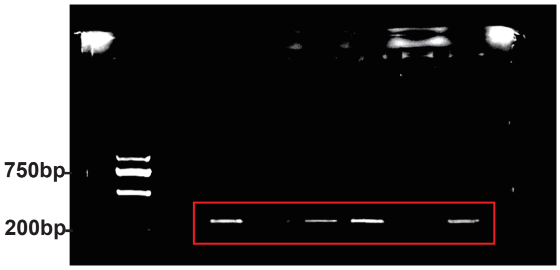

Fig.4A

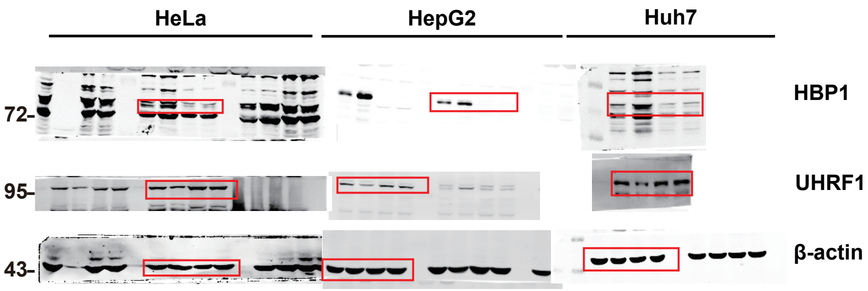

Fig.5C

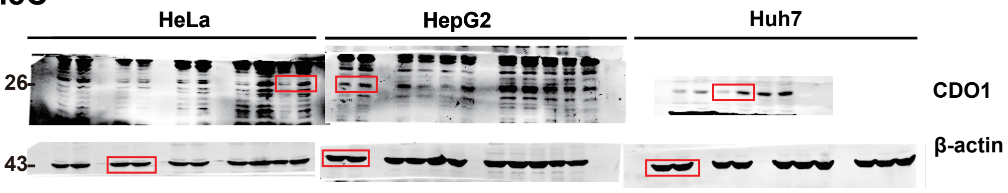

Fig.5D

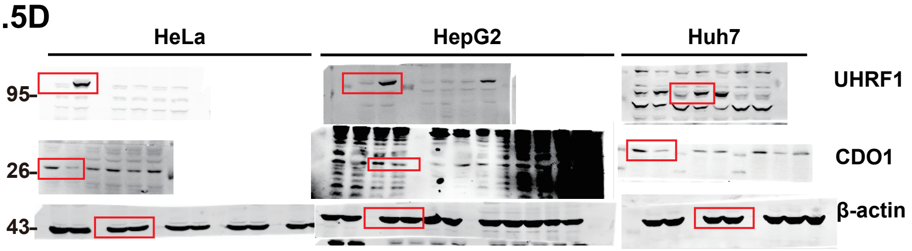

Fig.5E

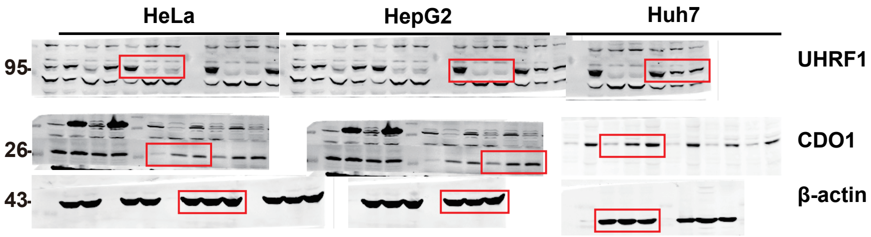

Fig.5H

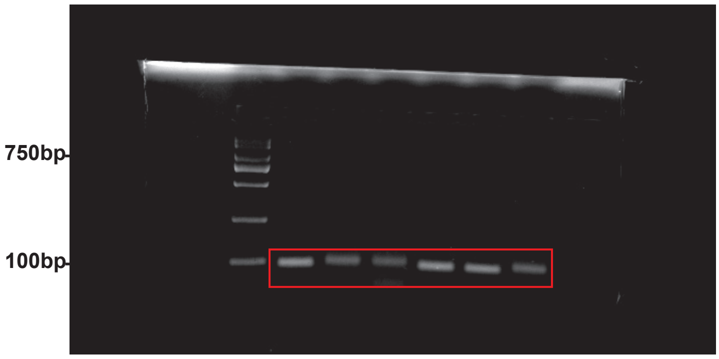

Fig.5K

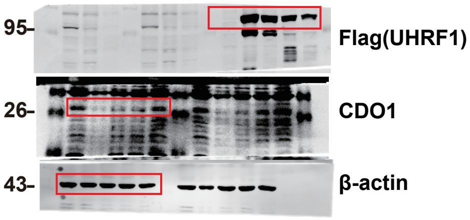

Fig.6E

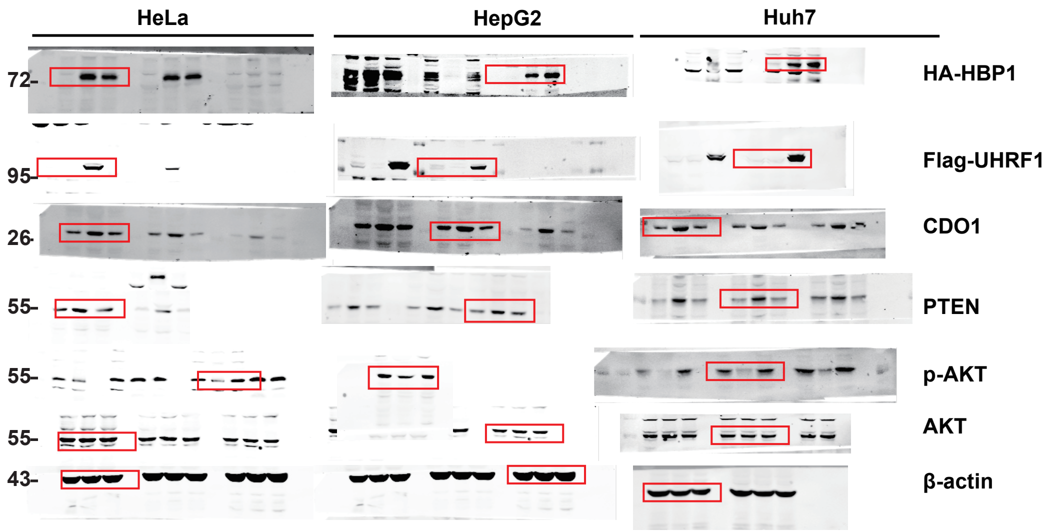
HeLa
HepG2
Huh7
72-
95-
26-
55-
55-
55-
43-
HA-HBP1
Flag-UHRF1
CDO1
PTEN
p-AKT
AKT
β-actin

Fig.6F

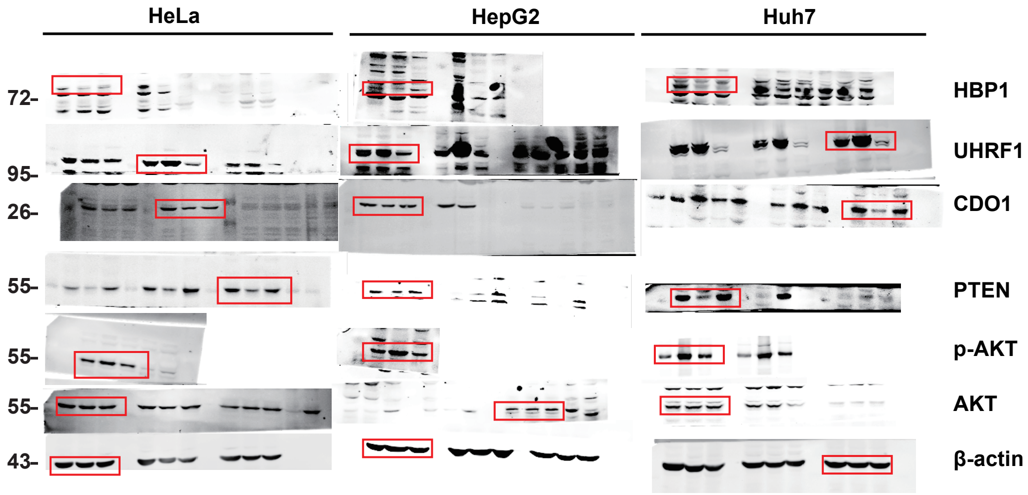
HeLa
HepG2
Huh7
72-
95-
26-
55-
55-
55-
43-
HBP1
UHRF1
CDO1
PTEN
p-AKT
AKT
β-actin

Fig.7E

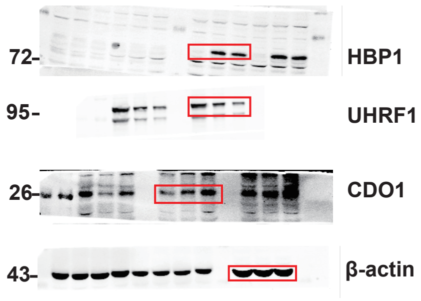

Fig.S2K

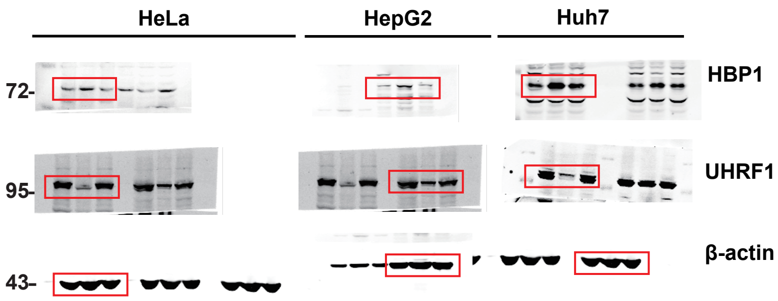

Fig.S5B

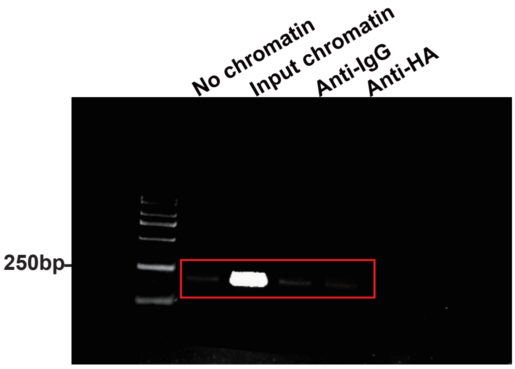

Fig.S6A

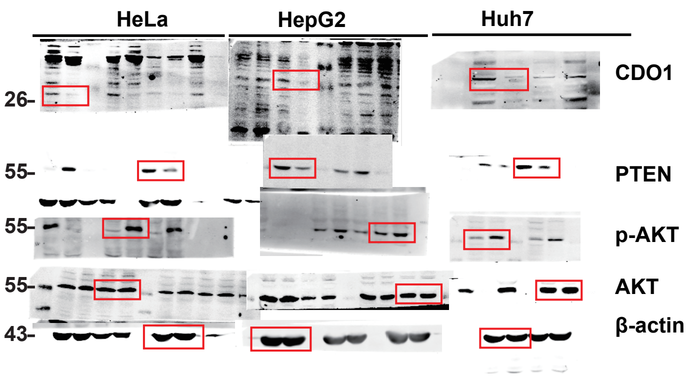

Fig.S6B

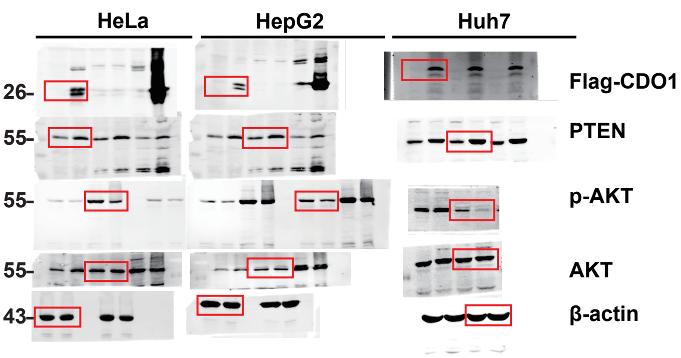

Fig.S7B

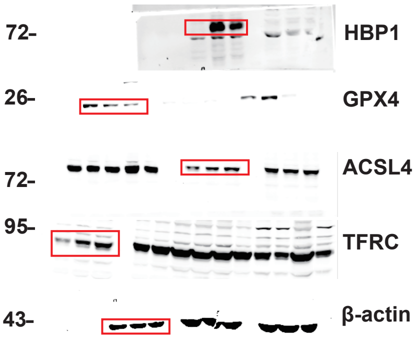

Supplement: S1 Raw Images — (PDF) [file pbio.3001862.s010.pdf]
