# Supplementary material for: The transcription factor HBP1 promotes ferroptosis in tumor cells by regulating the UHRF1-CDO1 axis
Source: PLoS Biol. 2023 Jul 5;21(7):e3001862. doi: 10.1371/journal.pbio.3001862 (PMC10351698; doi:10.1371/journal.pbio.3001862)
Supplement: Attachment — Submitted filename: Response letter.doc [file pbio.3001862.s011.doc]

RE: PBIOLOGY-D-22-02032R1 (The transcription factor HBP1 activates ferroptosis in tumor cells by regulating the UHRF1-CDO1 axis)

Dear Dr. Hodge,

Enclosed please find the revised manuscript entitled, “The transcription factor HBP1 activates ferroptosis in tumor cells by regulating the UHRF1-CDO1 axis” (PBIOLOGY-D-22-02032R1) that we wish to have considered for publication in PLOS Biology.

For our initial submission, the reviewers expressed enthusiasm towards our work. However, they expressed some concerns for the technical problems. In the past months, we have made major efforts to address these issues. We believe that we have addressed the most significant concerns. We have also vigorously adjusted the manuscript to PLOS Biology formal design.

I hope you will find our revised work acceptable for PLOS Biology in its current form. Your timely consideration of this manuscript is highly appreciated.

Sincerely,

Xiaowei Zhang, Ph.D.

Professor, Department of Biochemistry & Biophysics

Peking University Health Science Center

Beijing, China

Phone: 8610 8280 2418

Email: xiaoweizhang@bjmu.edu.cn

**Response to reviewers’ critique:**

In our initial submission of our manuscript **“**The transcription factor HBP1 activates ferroptosis in tumor cells by regulating the UHRF1-CDO1 axis” (PBIOLOGY-D-22-02032R1), the reviewers raised some concerns on the mechanisms of HBP1-HBP1-UHRF1-CDO1 axis in ferroptosis. In the past months, we have added additional data to address the concerns and revised the manuscript according to the reviewers’ suggestion. Those changes or corrections have been highlighted in blue in the revised manuscript. Given the space limit and the novelty of our work, we hope that our manuscript now merits publication in PLOS Biology in its current form.

Our point-by-point responses to the very thoughtful comments of the reviewers are outlined below.

**Reviewer #1:**

*In this manuscript, Yang and colleagues address that HBP1-UHRF1-CDO1 axis promotes the progression of ferroptosis and inhibits the proliferation of tumor cells and tumorigenesis. Ferroptosis induction to tumor suppression has been implicated as a new perspective for further research on anti-cancer therapy and holds great potential. The authors demonstrate that the transcription factor HBP1 represses UHRF1 expression by binding to an affinity site in the UHRF1 promoter through constructing YP191/192AA and YT478/479AA. They find that down-regulating the level of UHFR1 decreases the methylation level of the CDO1 promoter region and promotes CDO1 expression. As a result, the authors conclude that an increased level of CDO1 inhibits tumor cell proliferation through the activation of PTEN/PI3K/AKT signaling pathway because the key protein levels changed in CDO1 KO cell lines. The authors use real-time PCR, chromatin immunoprecipitation, immunohistochemistry staining, and transmission electron microscopy, demonstrating that HBP1-UHRF1-CDO1 axis leads to the deficiency of GSH synthesis, elevated lipid peroxidation and ferroptosis in tumor cells and generate the MPN-HBP1 nanodrugs to enhance the effect of tumor therapy. Overall, this is an interesting study, since the finding of HBP1-UHRF-CDO1 pathway gives us more insights into ferroptosis regulation by CDO1. However, the exact role of CDO1 in the regulation of tumor progression remains elusive.*

We appreciate your positive evaluation of our work. In response to your pertinent comments and suggestions, we have done some experiments and revised the manuscript carefully in an attempt to improve our work. The specific changes can be seen below.

*Major points:*

*1) Fer1 treatment blocked HBP1 upregulation by Ferroptosis-inducing agents (FINs) as shown in Fig.3E, does iron chelator like DFO also has the similar effect?*

We did Western blotting to test the effects of DFO on HBP1 and UHRF1 protein regulated by Erastin. As shown in S2K Fig, Erastin led to an increase in HBP1 protein level and a reduction in UHRF1 protein level, while DFO rescued the Erastin-induced changes in protein levels in the same way that Fer-1 did. This further suggests that HBP1-UHRF1 signaling pathway contributes to Erastin-induced ferroptosis (page 7, line 162-163).

*2) FINs suppressed proteasome-mediated HBP1 degradation as shown in Fig3 F-H. The authors need also check protein stability of HBP1 in the presence of FINs.*

We performed protein half-life experiment to examine protein stability of HBP1 in the presence of Erastin. As shown in Fig 3E and S3 Fig, Erastin clearly increased the half-life of HBP1 protein from 20.7 min to 53.5 min, indicating that FIN enhances HBP1 protein stability by inhibiting proteasome-mediated HBP1 degradation (page 8, line 170-171).

*3) CDO1 is known to reduce GSH and promote ferroptosis, the authors need to prove GSH level is the key to mediate HBP1-regulated ferroptosis using GSH synthesis inhibitor like BSO or supplementing GSH to restore the endogenous GSH level.*

We added Erastin to induce ferroptosis in HeLa, HepG2, and Huh7 cells overexpressing HBP1, and added exogenous glutathione (GSH). Cell viability was detected 24 h later. The results showed that exogenous GSH supplementation reversed the decline in cell viability induced by HBP1 (Fig 6K, left panel). Then we added Erastin to HeLa, HepG2 and Huh7 cells with HBP1 knockdown, followed by the addition of Butylamine-Sulfoximine-L (BSO) to inhibit glutathione synthesis. Finally, cell viability was detected. The results showed that BSO reversed the increase in cell viability caused by HBP1 knockdown (Fig 6K, right panel). Taken together, these data suggest that GSH level is the key to mediate HBP1-regulated ferroptosis (page 14, line 325-332).

*4) CDO1 also regulated PTEN/AKT signaling. Does this contribute to its regulation of ferroptosis, since AKT is known to regulate ferroptosis sensitivity through* *mTORC1 signaling.*

We verified that CDO1 inhibits the proliferation of tumor cells by regulating the PTEN/AKT signaling pathway (S6A-C Fig). In order to verify that in addition to directly inhibiting glutathione synthesis, CDO1 may also promote ferroptosis by regulating PTEN/AKT signaling. We added Erastin to induce ferroptosis in HeLa, HepG2 and Huh7 cells overexpressing CDO1, and added the PTEN inhibitor SF1670 (S6C Fig). The results showed that SF1670 could partially rescue the decrease of cell viability caused by overexpression of CDO1. This suggests that CDO1 may promote ferroptosis in part by regulating PTEN/AKT signaling (page 13, line 301-308).

*5) The authors need to prove the role of ferroptosis regulation by HBP1-UHRF-CDO1 in the tumor development via addition of ferroptosis inhibitor in vivo like Liprostatin-1. The current data couldn't demonstrate that HBP1-UHRF1-CDO1 axis suppresses tumor growth through promoting ferroptosis.*

We really appreciate your helpful advice. HepG2 cells with stable overexpression of HBP1 were injected subcutaneously in nude mice. After tumor development, nude mice were treated with intraperitoneal injection of the ferroptosis inhibitor Liprostatin-1. The results showed that Liprostatin-1 could reverse the inhibitory effect of HBP1 on tumor proliferation (Fig 6L). These results suggest that HBP1-UHRF1-CDO1 axis inhibits tumor growth by activating ferroptosis (page 14, line 338-342).

*6) Does HBP1 regulate ferroptosis and tumor growth solely through CDO1?*

We performed Realtime-PCR and ChIP experiments to test if HBP1 directly and specifically enhance *CDO1* gene transcription, thereby inducing ferroptosis. S5 Fig showed that although HBP1 increased CDO1 mRNA level and had no effect on the mRNA levels of other ferroptosis related genes (S5A Fig), HBP1 did not directly bind to the CDO1 promoter (S5B Fig), indicating that HBP1 transcriptionally enhance *CDO1* gene expression through UHRF1 in ferroptosis (page 12, line 288-291).

In addition, we constructed HepG2 cell lines that simultaneously knocks out CDO1 and overexpresses HBP1 to detect its cell viability (S6F Fig). The results showed that HBP1 enhanced Erastin-induced ferroptosis, and the induced ferroptosis could be rescued by Fer-1. However, when CDO1 was knocked out, the cell viability was mostly restored, but not completely recovered, indicating that HBP1 mainly induces ferroptosis through CDO1, but may also induce a small part of ferroptosis through other ways. HBP1 inhibits cell viability primarily by inhibiting glutathione synthesis (page 14, line 332-337).

*7) In Fig.7F, how about the impacts of PEI-HBP1 and MPN-HBP1 on UHRF and CDO1 expression? It seems that they also decrease GPX4 and increase ACSL4 and TFRC, the result of which could also promote ferroptosis independent of CDO1 and GSH. How to explain their effects?*

We performed Western blotting experiment to detect the effects of PEI-HBP1 and MPN-HBP1 on UHRF and CDO1 protein levels. As shown in Fig 7E, PEI-HBP1 and MPN-HBP1 reduced UHRF protein level and increased CDO1 protein level, and the effect of MPN-HBP1 was more significant, indicating that PEI-HBP1 and MPN-HBP1 could activate the UHRF1-CDO1 signaling pathway, and the effect of MPN-HBP1 was more obvious (page 16, line 375-376).

Yes, PEI-HBP1 and MPN-HBP1 also decreased GPX4 protein levels and increased ACSL4 and TFRC protein levels (S7B Fig) but had no effect on their mRNA levels (S5A Fig), indicating that HBP1 may regulate the expression of these proteins at the post-transcriptional level. To detect whether HBP1 promotes ferroptosis by other means than CDO1, we constructed HepG2 cell lines with both CDO1 knocked out and HBP1 overexpressed to detect cell viability (S6F Fig). The results showed that HBP1 induced ferroptosis mainly through CDO1, but a small part of ferroptosis may also be induced by other pathways. Therefore, we speculate that HBP1 may also promote ferroptosis by regulating GPX4, ACSL4, and TFRC protein levels. The specific regulation mechanism needs further study. We have added these to the discussion section (page 20, line 479-486).

**Reviewer #2:**

*"The transcription factor HBP1 activates ferroptosis in tumor cells by regulating the UHRF1-CDO1 axis". The manuscript proposes a pro-ferroptosis role of HBP1 by affecting the expression of UHRF1, which, in turn affect the CDO1 promoter methylation and expression. In addition, they have showed the iron-containing nano-particles with HBP1 can reduce the xenografts by triggering ferroptosis. While the proposed pathways have some novelties, there are some concerns about the biological context and quality of the experimental data.*

Thank you for your positive comments about our work. According to your pertinent comments and suggestions, we have done some experiments and revised the manuscript comprehensively in an attempt to improve the biological context and data quality of our work. The specific changes are shown below.

*Main critiques:*

*1. The main data on the effects of ferroptosis are quite modest. Given the strong phenotypes of ferroptosis, the proposed data are not compelling to support the hypothetic pathways.*

We appreciate your pointing out the defects of the data. We have repeated the experiment on the function of ferroptosis phenotype and added new experiments to improve the quality of the data. As shown in new Fig 4F and 4G, HeLa cells transfected with HBP1, HBP1+UHRF1 or shHBP1, shHBP1+shUHRF1 were treated with Erastin to detect the concentrations of Fe^2+^ (added experiments), MDA and GSH (repeated experiments). HBP1 overexpression combined with Erastin treatment significantly increased Fe^2+^ and MDA, and significantly decreased GSH in HeLa cells. HBP1 knockdown combined with Erastin treatment significantly decreased Fe^2+^ and MDA, and significantly increased GSH in HeLa cells. These HBP1 overexpression and knockdown effects can be reversed by UHRF1 overexpression and knockdown respectively. After statistical analysis, the data of each group showed significant differences, *p*<0.01. In addition, the ROS level in HepG2 cells after the above treatment was further detected by ROS fluorescence probe BODIPY-C11, and similar results were obtained (S4C Fig). Based on these sufficient experimental results, we have demonstrated favorably that HBP1 increases the sensitivity of tumor cells to ferroptosis by regulating the expression of UHRF1 (page 10, line 223-233, 242-245).

*2. Most of the knockdown experiments were done with one single shRNA without verifying by independent shRNAs or sgRNAs. The authors do not seem to be concerned about the potential off-target effects of these tools reported to have the potential for significant off-target effects.*

Thank you for pointing out the inaccuracy of shRNA construction. We constructed two shRNAs for HBP1 and two shRNAs for UHRF1 to prevent off-target effects and repeated some experiments with HBP1 or UHRF1 knockdown. As shown in Fig 1D, 3K, 3L, 5B, 5E, S2A-2G, the effects of both shRNAs are consistent, indicating that off-target effects can be excluded.

*3. The physiological function of the HBP1 was not known. In what context would this regulatory axis be relevant? No physiological or pathological contexts are provided.*

In order to explore the physiological function of HBP1, we used the data of TCGA database and GSEA analysis found that HBP1 was involved in the oxidative stress process of cells (S1B Fig). Next, we examined the effect of HBP1 on redox homeostasis of tumor cells, confirming that overexpression of HBP1 resulted in a significant decrease in NADPH/NADP^+^ and GSH/GSSG ratios (Fig. S2A), and knockdown of HBP1 resulted in an increase in the ratios (S2B Fig). This suggests that HBP1 can alter the redox homeostasis by transitioning to oxidation. Moreover, HBP1 increased the levels of total ROS and mitochondrial ROS in cells (S2C and S2D Fig) and increased the proportion of depolarized mitochondria in cells (S2E Fig), which predicted the decreased mitochondrial membrane potential (ΔΨm). Antioxidant NAC treatment restored the decreased ΔΨm caused by HBP1 overexpression. In addition, HBP1 reduced the antioxidant capacity of cells (S2F Fig) and made them more sensitive to H_2_O_2_-induced cell death (S2G Fig). These results indicate that HBP1 impairs the antioxidant capacity of tumor cells, disrupts the redox homeostasis of tumor cells, and makes tumor cells sensitive to oxidative stress-induced damage (page 6-7, line 117-147).

Next, using the CTRP website, we found that the expression of HBP1 was significantly correlated with the sensitivity of RSL-3 (S2H Fig). RSL-3, like Erastin, is an inducer of ferroptosis and can induce ferroptosis by disrupting the antioxidant capacity of tumor cells. Therefore, we speculate that HBP1 may be involved in the process of RSL-3 or Erastin-induced ferroptosis in the context that HBP1 can disrupt the antioxidant capacity of tumor cells (page 7, line 147-153).

In addition, we examined the role of the HBP1-UHRF1 axis in sorafenib-induced ferroptosis of HCC cells. As shown in S4 Fig, similar to Erastin, solafenib treated HepG2 cells with HBP1 overexpression, resulting in a further decline in cell viability (S4A Fig), a more significant increase in intracellular Fe^2+^, ROS and MDA levels (S4B-4D Fig), and a further decrease in GSH levels (S4D Fig). Overexpression of UHRF1 reversed these effects of HBP1. However, solafenib treated HepG2 cells with HBP1 knockdown caused the opposite of these changes, and UHRF1 knockdown rescued these effects caused by HBP1 knockdown (S4A-4C, S4E Fig). These data suggest that HBP1 also promotes the sensitivity of HCC cells to sorafenib-induced ferroptosis by inhibiting UHRF1 expression, thereby reducing the resistance of HCC cells to sorafenib (page 10-11, line 234-249).

*4. Most of the experiments were done in Hela and HepG2. Should the authors wish to study the relevance of the HCC, maybe more HCC cells should be used.*

Thank you for your valuable advice. We added another HCC cell line, Huh7, and repeated the experiments using the three cell lines of HeLa, HepG2 and Huh7 (Fig 1C, 1D, 3A-3C, 4A, 5C-5E, 6E, 6F, S6A-6E).
